# Detection of ATRX and IDH1-R132H immunohistochemistry in the progression of 211 paired gliomas

**DOI:** 10.18632/oncotarget.7650

**Published:** 2016-02-24

**Authors:** Jinquan Cai, Ping Zhu, Chuanbao Zhang, Qingbin Li, Zhiliang Wang, Guanzhang Li, Guangzhi Wang, Pei Yang, Jianlong Li, Bo Han, Chuanlu Jiang, Ying Sun, Tao Jiang

**Affiliations:** ^1^ Department of Neurosurgery, The Second Affiliated Hospital of Harbin Medical University, Harbin, China; ^2^ Beijing Neurosurgical Institute, Capital Medical University, Beijing, China; ^3^ Department of Neurosurgery, Beijing Tiantan Hospital, Capital Medical University, Beijing, China; ^4^ Department of Otolaryngology, Union Hospital, Tongji Medical College, Huazhong University of Science and Technology, Wuhan, China; ^5^ Beijing Institute for Brain Disorders Brain Tumor Center, Beijing, China; ^6^ China National Clinical Research Center for Neurological Diseases, Beijing, China; ^7^ Chinese Glioma Cooperative Group (CGCG), Beijing, China

**Keywords:** IDH1-R132H, ATRX loss, glioma, progression, evolution

## Abstract

Recurrence and progression to higher grade lesions are key biological events and characteristic behaviors in the evolution process of glioma. A small residual population of cells always escapes surgery and chemoradiation, resulting in a typically fatal tumor recurrence or progression. *IDH* mutation (isocitrate dehydrogenase) and ATRX (alpha-thalassemia/mental retardation, X-linked) loss/mutation occur in association and may represent early genetic alterations in the development of gliomas. However, their prognostic value in the evolution of gliomas still needs further investigation.

Two hundreds and eleven serial sampling of gliomas were included in our study. We used immunohistochemistry (IHC) to detect IDH1-R132H mutation and ATRX status and showed that the IDH1-R132H and (or) ATRX status could be necessary to provide the basic molecular information for the “integrated diagnosis” of gliomas. We illustrated an evaluation formula for the evolution of gliomas by IDH1-R132H combined with ATRX immunohistochemistry and identified the association of IDH1-R132H/ATRX loss accompanied by longer progression time interval of patients with gliomas. Furthermore, we observed that most recurrences had a consistent IDH1 and ATRX status with their matched primary tumors and demonstrated the progressive pattern of grade II astrocytoma/oligodendroglial tumors and anaplastic oligoastrocytoma with or without IDH1-R132H. Identification of IDH1-R132H and ATRX loss status in the primary-recurrent gliomas may aid in treatment strategy selection, therapeutic trial design, and clinical prognosis evaluation.

## INTRODUCTION

Diffuse gliomas are classified into astrocytomas, oligoastrocytomas and oligodendrogliomas of grade II, grade III and glioblastoma based on the 2007 World Health Organization (WHO) Classification of Tumors of the Central Nervous System [1]. Although patients with low grade gliomas (LGGs, grade II) [2] have a more favorable prognosis than patients with high grade gliomas (grade III and IV), in 50–75% of patients with low grade gliomas, the tumors grow continuously and tend to progress to a higher grade, leading to neurological disability and ultimately to death [3].

The mutations of *IDH* and *ATRX* occur in early stage of gliomagenesis and characterize specific subtypes of gliomas in adults [4, 5]. The majority of oncogenic *IDH1* mutations are heterozygous missense mutations with a change of guanine to adenine at position 395 (G395A), leading to the replacement of arginine by histidine at codon 132 (IDH1-R132H) at the enzymatic active site [6, 7]. *ATRX* mutations or loss, companied by an alternative lengthening of telomeres (ALT) phenotype, impacted biological behaviors of astrocytic tumor cells, associated with favorable survival of patients with astrocytic tumors [8, 9].

According to the “ISN-Haarlem” consensus [10], the “integrated” diagnosis was recommended based on histology and stepwise analysis with initial immunohistochemistry for ATRX and IDH1-R132H followed by chromosome 1p/19q status analysis and *IDH* sequencing [11]. In this study, we collected 211 serial sampling of gliomas and detected ATRX and IDH1-R132H status in the progression of gliomas by immunohistochemistry. The result will help to evaluate the progressive pattern and time interval of patients with the initial gliomas using the reference histology combined with IDH1-R132H and ATRX status.

## RESULTS

### Frequency and diagnostic value of IDH1-R132H and ATRX loss in gliomas

To test the impact of IDH1-R132H and ATRX on routine diagnostic neuropathology and their evaluation for progression of gliomas, we analyzed a series of 211 serial sampling of glioma tissues, including 103 astrocytomas (A, AA), 25 oligodendrogliomas (O, AO), 123 oligoastrocytomas (OA, AOA) and 181 glioblastomas (pGBM, sGBM, rGBM) by immunohistochemistry for IDH1-R132H and ATRX evaluation.

**Figure 1:**
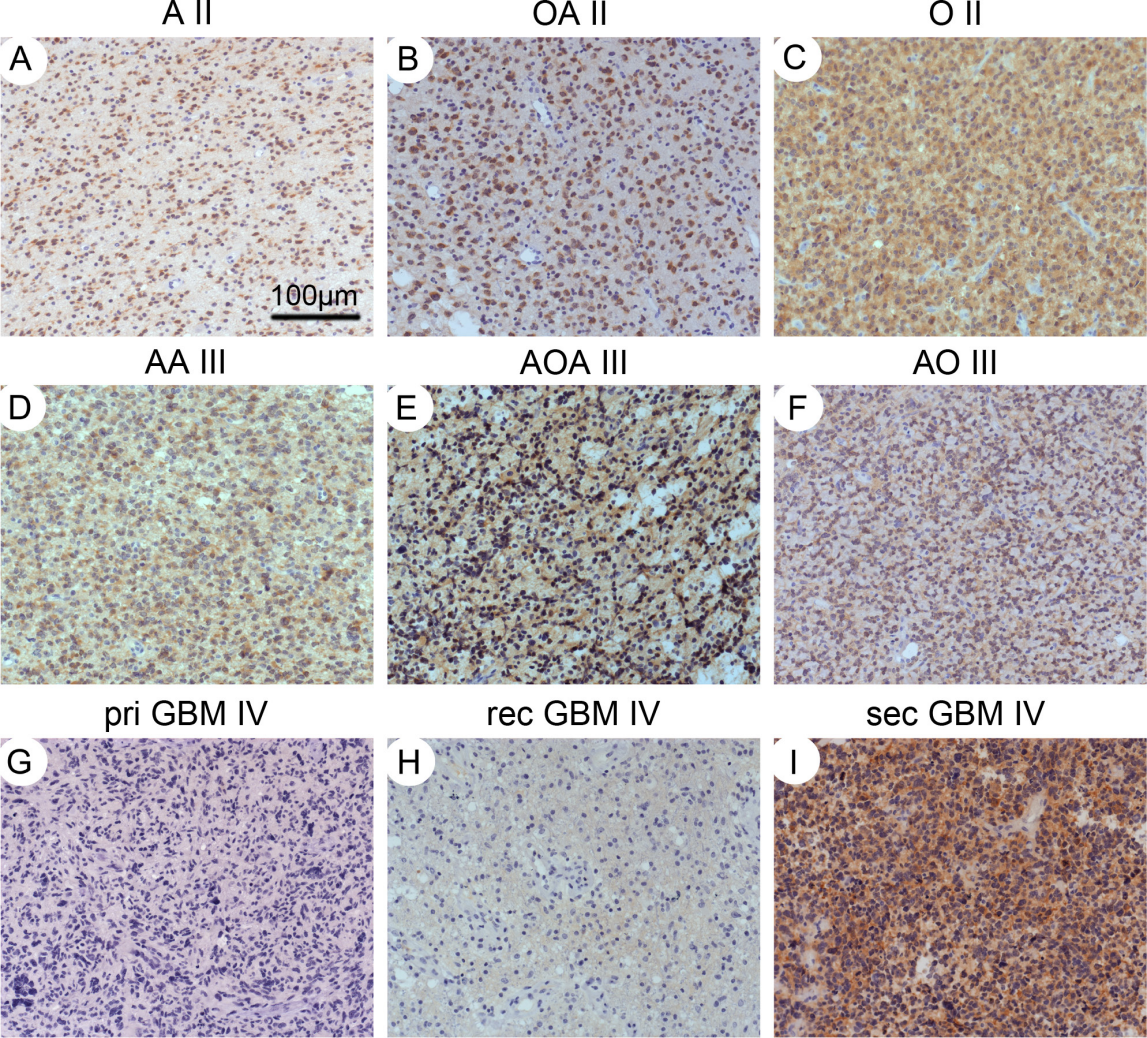
IHC staining for IDH1-R132H Diffuse astrocytomas (**A**), oligoastrocytoma (**B**) and oligodendroglioma (**C**) with the IDH1-R132H positive tumor cells. Anaplastic astrocytomas (**D**), oligoastrocytoma (**E**) and oligodendroglioma (**F**) with the positive IDH1-R132Htumor cells. Primary (**G**) and recurrent glioblastoma (**H**) showed negative for H09, however, secondary glioblastoma showed the strongly positive tumor cells (**I**). Magnification: ×100.

IDH1-R132H staining positive and negative scoring was unequivocal. The majority of positive cases demonstrated a strong perinuclear cytoplasmic staining with additional weaker nuclear staining (Figure 1). In our series of 64 A II, 37 tumors were scored positive (57.81%, Figure 1A). There was an even higher rate of positive cases in OA II (40/49, 81.63%, Figure 1B) and O II (9/12, 75%, Figure 1C) labeled by H09. Rates for AA III, AOA III and AO III were also high, with 13 of 27 AA III (48.15%, Figure 1D), 38 of 68 AOA III (55.88%, Figure 1E) and 5 of 9 AO III (55.56%, Figure 1F) positive for H09. Among 114 pGBM, 17 positive cases were detected (14.91%, Figure 1G, 1H), while 40 of 59 sGBM (67.8%, Figure 1I) bound IDH1-R132H (Table 1, *p* = 0.009, Fisher's exact test).

**Table 1:** Frequency of IDH1-R132H and ATRX loss in gliomas

Histology	IDH			ATRX			IDH_R132H+ATRX_loss		
	total	R132H	frequency	total	loss	frequency	R132H	loss	frequency
A	64	37	57.81	64	49	76.56	37	34	91.89
AA	27	13	48.15	27	21	77.78	13	10	76.92
O	12	9	75.00	12	1	8.33	9	1	11.11
AO	9	5	55.56	9	1	11.11	5	1	20.00
OA	49	40	81.63	49	23	46.94	40	21	52.50
AOA	68	38	55.88	68	20	29.41	38	15	39.47
pGBM	114	17	14.91	114	14	12.28	17	5	29.41
sGBM	59	40	67.80	59	45	76.27	40	36	90.00

**Abbreviations:** A, astrocytoma; AA, anaplastic astrocytoma; O, oligodendroglioma; AO, anaplastic oligodendroglioma; OA, oligoastrocytoma; AOA, anaplastic oligoastrocytoma; pGBM, primary glioblastoma; sGBM, secondary glioblastoma; IDH, isocitrate dehydrogenase, ATRX, alpha-thalassemia/mental retardation, X-linked.

**Figure 2:**
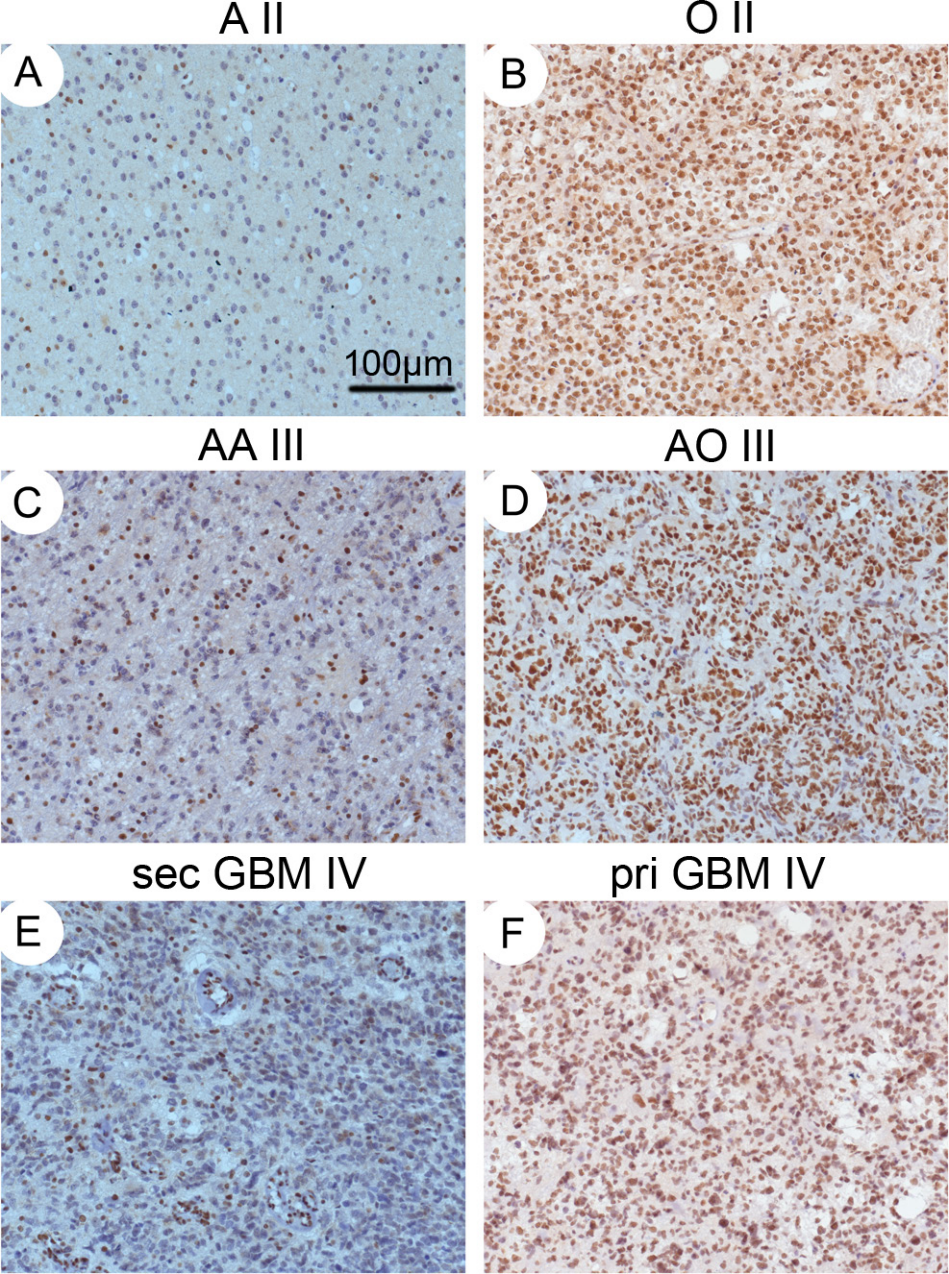
IHC staining for ATRX Diffuse (**A**), anaplastic astrocytomas (**C**) and secondary glioblastoma (**E**) with loss of ATRX staining in tumor cell nuclei. Oligodendroglioma (**B**), anaplastic oligodendroglioma (**D**) and primary glioblastoma (**F**) with strong nuclear ATRX staining. The endothelial cells are always positive, serving as controls. Magnification: ×100.

ATRX immunohistochemistry was positive in 174 of 402 cases with a strong nuclear immune reaction in most cases (Figure 2). Loss of ATRX was evident in 228 of the 402 cases with the bulk of tumor cells negative for ATRX, whereas endothelia and infiltrating inflammatory cells as well as residual neurons retained ATRX expression. As reported earlier, the frequency of ATRX loss was higher in A, AA and sGBM (A, 49/64, 76.56%; AA, 21/27, 77.78%; sGBM, 45/59, 76.27%, Figure 2A, 2C, 2E) than in OA, AOA (OA, 23/49, 46.94%; AOA, 20/68, 29.41%) and low in O, AO and pGBM (O, 1/12, 8.33%; AO, 1/9, 11.11%; pGBM, 14/114, 12.28%, Table 1, *p* = 0.009, Fisher's exact test). Our results revealed that IDH1-R132H dominated in grade II/III gliomas and secondary GBM. ATRX loss predominantly occurred in grade II/III astrocytoma and secondary GBM. These two events primarily co-occurred grade II/III astrocytoma and secondary GBM. In 30 cases, no immunoreaction in the entire tissue or specific patterns of immunoexpression in proximity to necrosis were observed, these cases were not scored and consequently not considered for statistical evaluation.

**Figure 3:**
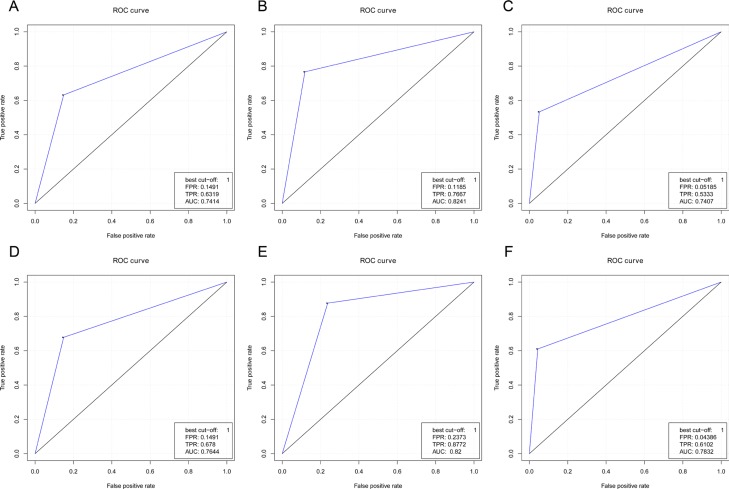
ROC curves of using IDH1-R132H and (or) ATRX status in discriminating the classification of glioma histology The AUC of using IDH1-R132H as diagnostic biomarkers for discriminating between primary GBM and grade II/III glioma, secondary GBM is 0.7414 (the sensitivity is 63.19%, the specificity is 85.09%, (**A**). The AUC of using ATRX loss as diagnostic biomarkers for discriminating between primary GBM, oligodendroglioma (WHO grade II/III) and secondary GBM, astrocytoma (WHO grade II/III) is 0.8241 (the sensitivity is 76.67%, the specificity is 88.15%, (**B**). When IDH1-R132H combined with ATRX loss serves as diagnostic biomarkers for discriminating between astrocytoma (WHO grade II/III), secondary GBM, and primary GBM, oligodendroglioma (WHO grade II/III), the AUC is 0.7407 (the sensitivity is 53.33%, the specificity is 94.815%, (**C**).

Then we performed receiver operating characteristic (ROC) analysis to delineate the sensitivity and specificity of IDH1-R132H and (or) ATRX loss for WHO histology classification. As shown in Figure 3, the area under roc curve (AUC) of using IDH1-R132H as a diagnostic biomarker for discriminating between pGBM and grade II/III glioma, sGBM was 0.7414 (sensitivity 63.19%, specificity 85.09%, Figure 3A). The AUC of using ATRX loss as a diagnostic biomarker for discriminating between pGBM, oligodendroglioma (WHO grade II/III) and sGBM, astrocytoma (WHO grade II/III) was 0.8241 (sensitivity 76.67%, specificity 88.15%, Figure 3B). When combining IDH1-R132H with ATRX loss as diagnostic biomarkers for discriminating between pGBM, oligodendroglioma (WHO grade II/III) and sGBM, astrocytoma (WHO grade II/III), the AUC was 0.7407 (sensitivity 53.33%, specificity 94.815%, Figure 3C). We also performed ROC analysis for distinguishing primary and secondary GBMs using IDH1-R132H and (or) ATRX. When we just used IDH1-R132H status for ROC analysis, the AUC is 0.7644 (the sensitivity is 67.8%, the specificity is 85.09%, Figure 3D). When we just used ATRX loss status for ROC analysis, the AUC is 0.82 (the sensitivity is 87.72%, the specificity is 76.27%, Figure 3E). When we combined IDH1-R132H and ATRX loss status for ROC analysis, the AUC is 0.7832 (the sensitivity is 61.02%, the specificity is 95.61%, Figure 3F). These results indicated that IDH1-R132H and (or) ATRX loss status might have a good predictive ability for WHO histology diagnosis.

### Prognostic value of IDH1-R132H and ATRX loss in the evolution of gliomas

We analyzed the cohort of patients with primary and recurrent gliomas to observe the value of IDH1-R132H or ATRX as biomarkers evaluating the progression-free survival of patients with gliomas. In our cohort, we estimated the progression-free survival (PFS) in WHO grade II-IV patients and observed a remarkable separation (Median PFS of low grade gliomas, LGGs = 830 days, Median PFS of anaplastic gliomas, AGs = 495 days, Median PFS of GBMs = 394 days, *p* < 0.0001, log-rank test, Table 2).

**Table 2:** Analysis of the progression-free survival based on the WHO histology and grade of gliomas

Histology	total	Median PFS (days)	1-year PFS survival rate	2-year PFS survival rate	3-year PFS survival rate	4-year PFS survival rate	5-year PFS survival rate	*p* value
LGG	100	830	84	55	27	13	7	< 0.0001
pA	50	693	82	50	28	14	8	
pO	9	721	67	44	22	22	11	
pOA	41	860	90	63	27	10	5	
AG	47	495	68	32	15	6		
pAA	10	611	70	50	20	10		
pAO	5	792	100	60	40			
pAOA	32	398	63	22	9	6		
pGBM	64	394	52	16	6	2	2	

**Abbreviations:** WHO, World Health Organization; PFS, progression-free survival; LGG, low grade glioma; pA, primary astrocytoma; pO, primary oligodendroglioma; pOA, primary oligoastrocytoma; AG, anaplastic glioma; pAA, primary anaplastic astrocytoma; pAO, primary anaplastic oligodendroglioma; pAOA, primary anaplastic oligoastrocytoma; pGBM, primary glioblastoma.

**Figure 4:**
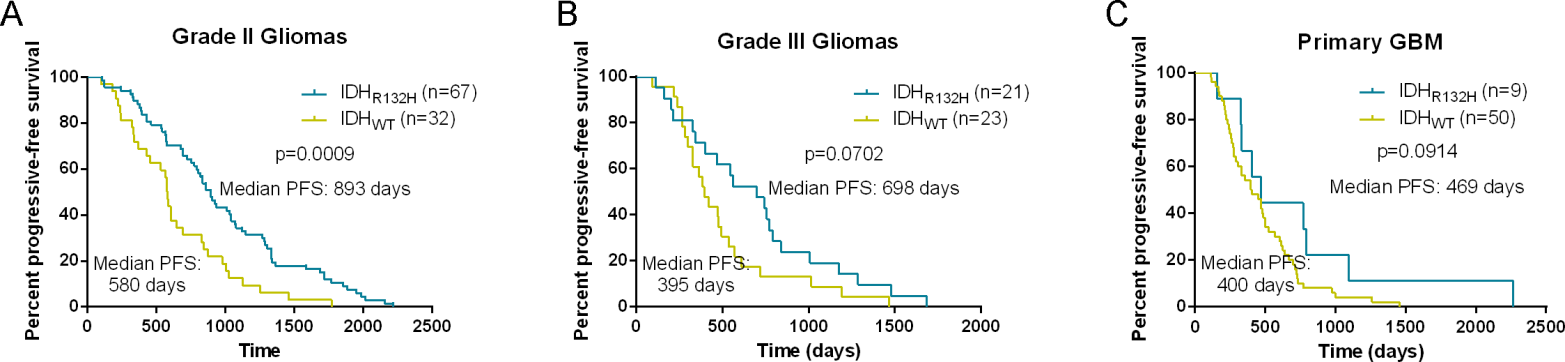
Kaplan-Meier analysis of progression-free survival in patients with IDH1-R132H and not Progression-free survival was significantly longer in patients with grade II gliomas harboring IDH1-R132H than in patients with IDH1 wild-type tumors (**A**). Although PFS was longer in patients with anaplastic gliomas or primary GBM harboring IDH1-mutated tumors than in patients with IDH1 wild-type tumors, this difference was not significant (**B**) and (**C**).

**Figure 5:**
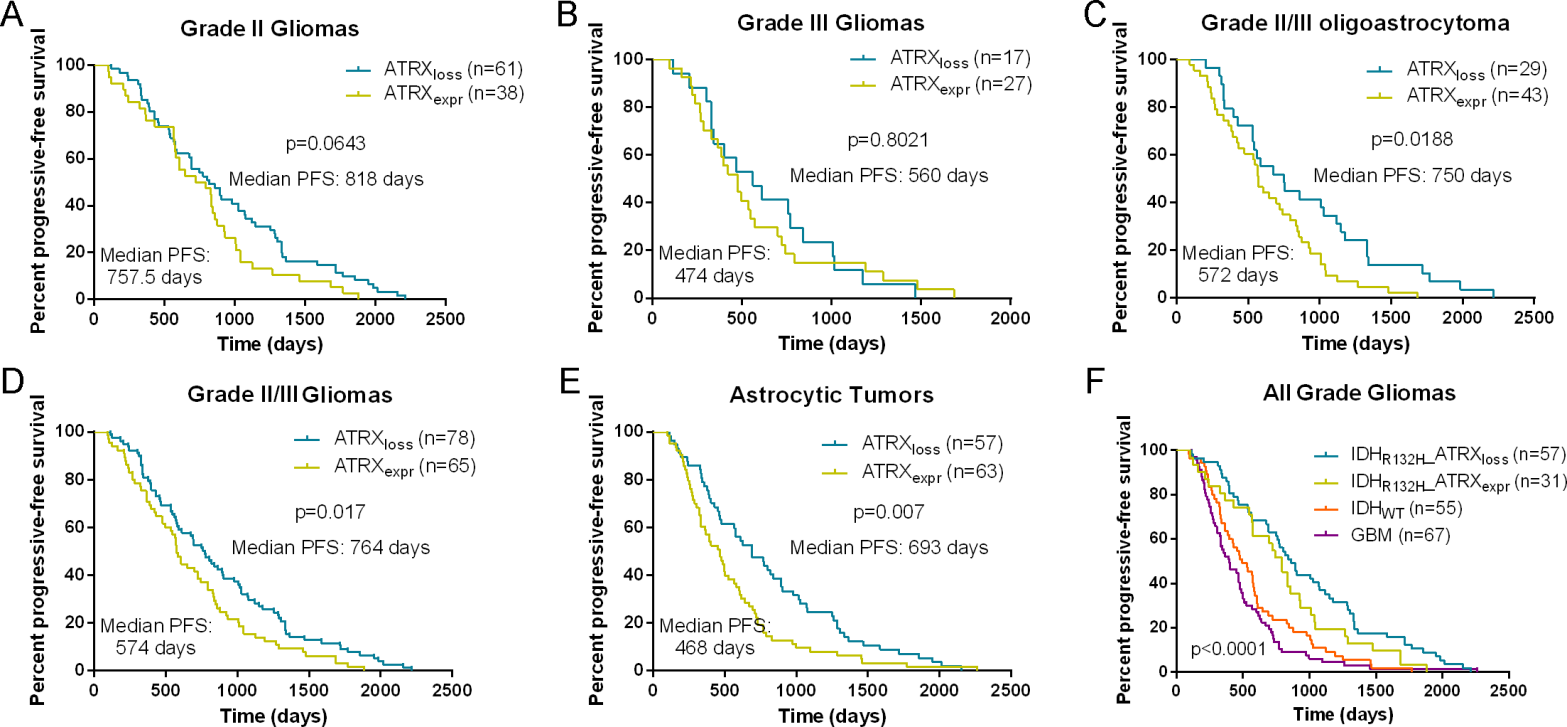
Kaplan-Meier analysis of progression-free survival in patients with ATRX protein loss and expression In the cohort of patients with grade II or grade III tumors (**A**) and (**B**), patients with ATRX loss had a trend for longer PFS compared with those with ATRX expression. ATRX loss was associated with longer progression-free survival in patients with grade II/III oligoastrocytomas or grade II/III gliomas (**C**) and (**D**). Patients with astrocytic tumors losing ATRX had a longer progression-free survival than patients with tumors expressing ATRX (**E**). In additions, there was a trend that patients with IDH1-R132H and ATRX loss grade II/III gliomas had longer PFS than patients with IDH1-R132H and ATRX positive grade II/III gliomas and PFS of patients with GBM was similar to that of patients with IDH wild type (**F**).

PFS was significantly longer in patients with grade II gliomas harboring IDH1-R132H than in patients with IDH1 wild-type tumors (Median PFS of IDH1-R132H = 893 days, Median PFS of IDH1-wt = 580 days, *p* = 0.0009, log-rank test; Figure 4A). Although PFS was longer in patients with AG or pGBM harboring IDH1-mutated tumors than in patients with IDH1 wild-type tumors, this difference was not significant (*p* = 0.0702 and *p* = 0.0914, respectively, log-rank test; Figure 4B, 4C). Although there was no significant difference between ATRX loss and ATRX expression in the cohort of patients with grade II or grade III tumors (Figure 5A, 5B), ATRX loss was associated with longer progression-free survival in patients with grade II/III oligoastrocytomas or grade II/III gliomas (Figure 5C, 5D). Patients with astrocytic tumors losing ATRX had a longer progression-free survival than patients with tumors expressing ATRX (Figure 5E). In additions, there was a trend that patients with IDH1-R132H and ATRX loss grade II/III gliomas had longer PFS than patients with IDH1-R132H and ATRX positive grade II/III gliomas and PFS of patients with GBM was similar to that of patients with IDH wild type (Figure 5F). We have demonstrated the basic clinical characteristics among the IDH-R132H/ATRXloss, IDH-R132H/ATRXexpr, IDH-WT and GBM groups in the Supplementary Table1. After incorporation of age, WHO grade, IDH1 and ATRX status into the multivariate model, the prognostic value of IDH1-R132H was still significant (Table 3).

**Table 3:** Cox regression analysis for the progression-free survival of patients with gliomas

	Univariate Cox Model			Mutivariate Cox Model		
	Hazard ratio	95%CI	*p* value	Hazard ratio	95%CI	*p* value
Age ≤ 45 vs. > 45	0.703	0.523–0.946	0.02	0.89	0.509–1.03	0.474
Male vs. Female	0.906	0.686–1.197	0.488			
Astrocytic vs. oligodendroglial	1.123	0.849–1.486	0.417			
IDH1-R132H positive vs. negative	0.437	0.326–0.586	3.15E–08	0.517	0.369–0.723	1.19E–04
ATRX loss vs. expression	0.645	0.486–0.856	0.002	0.913	0.659–1.264	0.584
WHO grade II/III vs. IV	0.529	0.388–0.772	5.79E–05	0.724	0.509–1.030	0.072

**Abbreviations:** WHO, World Health Organization; PFS, progression-free survival; LGG, low grade glioma; pA, primary astrocytoma; pO, primary oligodendroglioma; pOA, primary oligoastrocytoma; AG, anaplastic glioma; pAA, primary anaplastic astrocytoma; pAO, primary anaplastic oligodendroglioma; pAOA, primary anaplastic oligoastrocytoma; pGBM, primary glioblastoma; IDH, isocitrate dehydrogenase, ATRX, alpha-thalassemia/mental retardation, X-linked.

### IDH1-R132H and ATRX loss in matched primary and recurrent gliomas

**Figure 6:**
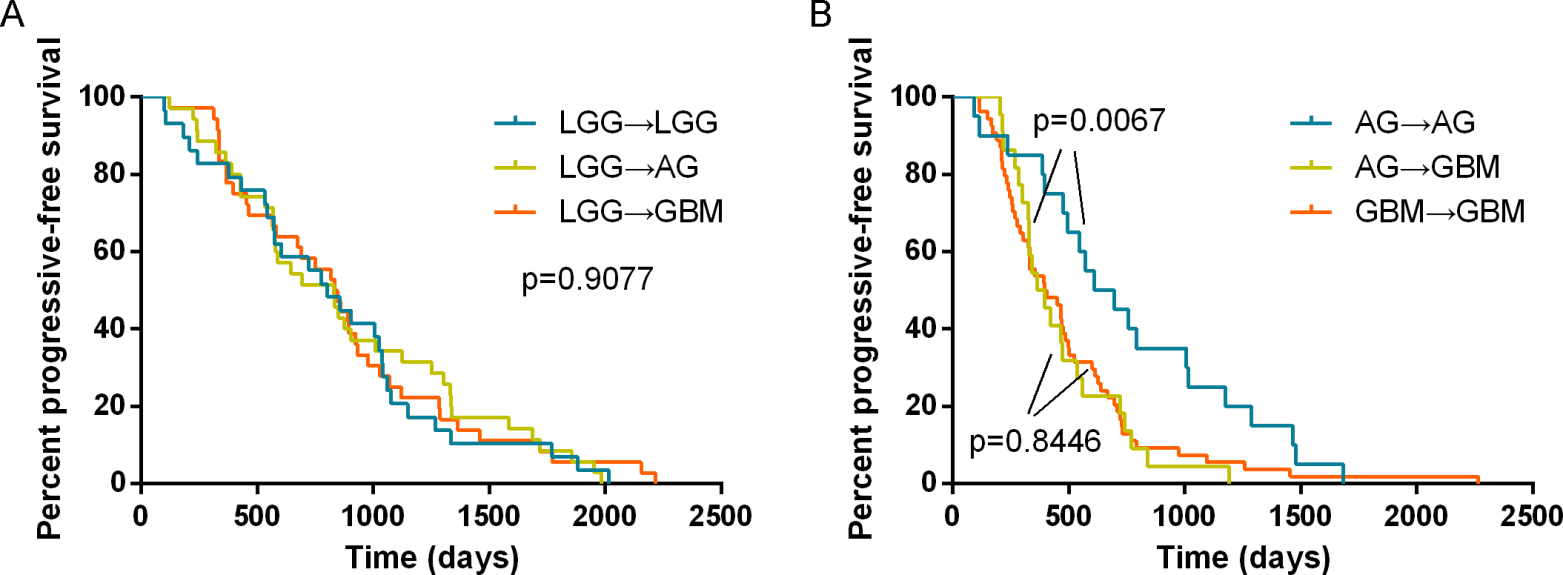
Analysis of the progression-free survival based on the different evolution patterns of gliomas There was no difference in the progression-free survival when LGG malignantly recurred to LGG, AG or GBM (Median PFS = 802 days, 830 days and 835 days, respectively, (**A**). However, the time interval of AG to GBM was significantly shorter than that of AG to AG (Median PFS = 365 days and 611 days, respectively, *p* = 0.0067, log-rank test). The progressive time interval of GBM to GBM was similar to that of AG to GBM (Median PFS = 395 days, *p* = 0.8446, (**B**).

First, we excluded these pairs that the primary gliomas were high grade tumor while the recurrence was lower grade tumor and then analyzed the pattern of glioma progression. As shown in Table 4, the recurrent tumors of primary LGGs included LGGs, AGs and GBMs of which rates corresponded to 29% (29/100), 35% (35/100) and 36% (36/100), respectively. The time interval of the three progressive patterns was analyzed. There was no difference in the progression-free survival when LGG malignantly recurred to LGG, AG or GBM (Median PFS = 802 days, 830 days and 835 days, respectively, Figure 6A). However, the time interval of AG to GBM was significantly shorter than that of AG to AG (Median PFS = 365 days and 611 days, respectively, *p* = 0.0067, log-rank test). The progressive time interval of GBM to GBM was similar to that of AG to GBM (Median PFS = 395 days, *p* = 0.8446, Figure 6B). IDH1-R132H occurred stably in the primary tumors and matched recurrent tumors, although not all the recurrent tumors showed consistent IDH1-R132H status as the corresponding primary tumors (Table 4). Because there was a too high or too low frequency of ATRX loss occurrence in the astrocytoma (WHO II/III) or oligodendroglioma (WHO II/III) and pGBM, the rate of ATRX loss was not observed between primary and matched recurrent tumors. After combining the IDH1-R132H and ATRX loss status, we also illustrated that most recurrences had a consistent IDH1 and ATRX status with their primary tumors (IDH1-R132H/ATRXloss: 43/59, 74.1%, *p* < 0.0001; IDH1-R132H/ATRXexpr: 18/36, 50%, *p* < 0.0001; IDH-WT: 77/102, 75.5%, *p* < 0.0001, Table 5).

**Table 4:** Analysis of the progression-free survival based on the different evolution patterns of gliomas

Histology	total	Median PFS (days)	IDH status in initial tumors	IDH status in relapse tumors	*p* value
LGG→LGG	29	802	20/27	20/28	
LGG→AG	35	830	20/34	21/34	
LGG→GBM	36	835	26/35	28/35	
AG→AG	20	611	10/20	12/20	0.0067
AG→GBM	22	365	10/22	10/22	
GBM→GBM	54	395	8/53	8/52	

**Abbreviations:** PFS, progression-free survival; LGG, low grade glioma; AG, anaplastic glioma; GBM, glioblastoma; IDH, isocitrate dehydrogenase, ATRX, alpha-thalassemia/mental retardation, X-linked.

**Table 5:** Frequency of IDH1-R132H and ATRX loss in matched primary and recurrent gliomas

Initial tumors	total	Median PFS (days)	Recurrent tumors				*p* value
			IDHmut	IDHwt	IDHmut_ATRXloss	IDHmut_ATRXexpr	
IDHmut_ATRXloss	59	839	78 (82%)	17	43 (74.1%)	8	< 0.0001
IDHmut_ATRXexpr	36	770			8	18 (50%)	< 0.0001
IDHwt	102	455	25	77 (75.5%)	12	13	< 0.0001

**Abbreviations:** mut, mutation; wt, wild type; expr, expression; IDH, isocitrate dehydrogenase, ATRX, alpha-thalassemia/mental retardation, X-linked.

### Progressive pattern of primary glioma with or without IDH1-R132H

**Figure 7:**
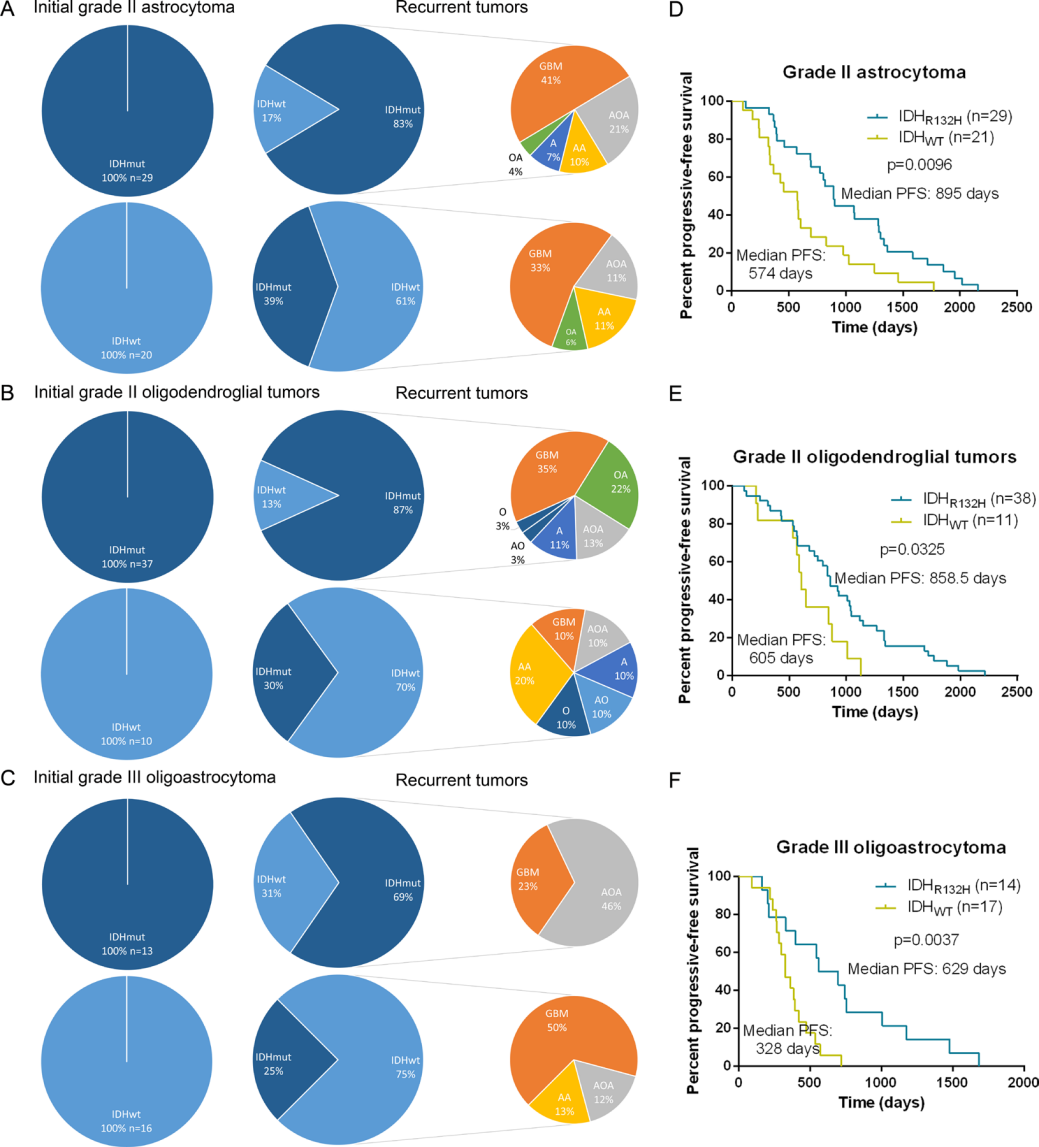
Progressive pattern of primary glioma with or without IDH1-R132H Recurrences from initial grade II astrocytomas always referred to secondary GBM, anaplastic oligoastrocytomas and anaplastic astrocytomas regardless of with or without IDH1-R132H (**A**). Recurrences from IDH1-R132H grade II oligodendroglial tumors mainly showed secondary glioblastomas, oligoastrocytomas morphology. In contrast, there were no main histology characteristics in the recurrences from the initial IDH1-WT grade II oligodendroglial tumors (**B**). Glioblastomas and anaplastic oligoastrocytomas were occupied on the most percentage of relapses from primary anaplastic oligoastrocytomas. However, most of relapses from IDH1-R132H AOA were recurrent anaplastic oligoastrocytomas; most of relapses from IDH1-WT AOA were secondary GBM (**C**). Patients with IDH1-R132H tumors had a longer progression-free survival than patients with IDH1-WT tumors, irrespective of histology (**D**, **E** and **F**).

Generally, diffuse LGGs malignantly transformed into AGs, followed by secondary GBM, but occasionally, there might be no evidence of the former one. Meanwhile, some recurrences had different morphology from initial tumors (astrocytic to oligodendroglial and vice versa). In our cohort, we discriminated the IDH1-R132H initial tumors from the IDH1-WT tumors and analyzed the evolution pattern of 125 primary-recurrent paired tumors, 49 recurrences of which were originated from grade II A (Figure 7A), 47 were from grade II O (Figure 7B) and 29 were grade III AOA (Figure 7C). We observed that regardless of with or without IDH1-R132H, the histology of recurrences from initial grade II A could be sGBM, AOA and AA. Although with the similar evolution pattern, patients with IDH1-R132H grade II A had a longer progressive time interval compared with patients with IDH1-WT grade II A (Median PFS = 895 days, Median PFS = 574 days, respectively, *p* = 0.0096, Figure 7D). Recurrences from IDH1-R132H grade II O mainly showed sGBM, OA morphology. In contrast, there were no main histology characteristics in the recurrences from the initial IDH1-WT grade II O. Patients with IDH1-R132H grade II O had a longer progression-free survival than patients with IDH1-WT grade II O (Median PFS = 858.5 days, Median PFS = 605 days, respectively, *p* = 0.0325, Figure 7E). GBM and AOA were the main recurrent histology from primary AOA. However, most of relapses from IDH1-R132H AOA were recurrent AOA; most of relapses from IDH1-WT AOA were sGBM. Patients with IDH1-R132H AOA also had a longer progression-free survival than patients with IDH1-WT ones (Median PFS = 629 days, Median PFS = 328 days, respectively, *p* = 0.0037, Figure 7F).

## DISCUSSION

It is accepted that *IDH* mutation and *ATRX* mutation were used as diagnostic and prognostic biomarkers for molecular classification of gliomas [12, 13]. However, their prognostic value for glioma progression is not clear, especially ATRX loss.

In our study, we used immunohistochemistry to detect IDH1-R132H and ATRX status instead of sequencing, aiming to provide a mature and simple tool for clinical practice. In the light of the “ISN-Haarlem” consensus, the routine approach to all diffuse astrocytic and oligodendroglial gliomas begins with performing IHC for ATRX and IDH1-R132H expression. Our results showed that the IDH1-R132H and (or) ATRX loss status could be necessary to provide the basic molecular information for the “integrated diagnosis” of gliomas.

*IDH* mutation is thought to be an early if not the initial event in the development of low-grade astrocytomas and oligodendrogliomas. In support of this hypothesis, *IDH* mutation is found in secondary glioblastomas but rare in primary glioblastomas [6]. *IDH1* mutations have been shown to alter the enzymatic activity of the encoded protein, leading to up-regulation of hypoxia inducible factor-1α (HIF-1α) [14, 15]. HIF-1α plays an important role in the process of angiogenesis while also supporting tumor cell survival and proliferation [14, 16]. Although the properties of the *IDH1* mutation would promote tumor growth, *IDH* mutation commonly indicates a favorable prognosis independent of WHO grades [7, 17–19].

Mutations and loss of expression of alphathalassemia/mental retardation syndrome X-linked (*ATRX*) was first reported in pancreatic neuroendocrine tumors [20]. ATRX protein plays a variety of key roles at tandem repeat sequences within the genome, including prevention of replication fork stalling, the deposition of a histone variant, and the suppression of a homologous recombination-based pathway of telomere maintenance [9]. Recently, mutation/loss of *ATRX* was identified as a potent biomarker in grade II-III gliomas and was associated with recurrent ones [21]. *ATRX* gene mutations were significantly correlated with ALT positivity [22]. Loss of ATRX in ALT frees macroH2A1.1 to bind and sequester tankyrase 1, thereby preventing resolution of sister telomere cohesion. Forced resolution of sister telomere cohesion induces excessive recombination between non-homologs, genomic instability, and impaired cell growth, indicating the *ATRX*-macroH2A1. 1-tankyrase axis as a potential therapeutic target in ALT tumors [23]. Inhibition of the protein kinase ATR, a critical regulator of recombination recruited by RPA, disrupts ALT and triggers chromosome fragmentation and apoptosis in ALT cells. The cell death induced by ATR inhibitors is highly selective for cancer cells that rely on ALT, suggesting that such inhibitors may be useful for treatment of ALT-positive cancers [24]. In patients with 1p/19q noncodeleted tumors with *IDH* mutations, those who were ATRX positive might have benefitted more than those who were negative from pre-RT PCV [25]. Whether these noncodeleted ATRX-positive tumors are biologically identical to codeleted tumors, save for absence of visible loss of 1p/19q, or represent laboratory errors in codeletion testing is unknown.

We previously reported that reduced expression of ATRX inhibited the migration, promoted apoptosis and reduced proliferation of glioma cells, suggesting the important role of ATRX in gliomas. Similarly, a report depicted how loss of ATRX could affect neuroprogenitor cell apoptosis [26].

IDH1-R132H and ATRX expression status are essential to the diagnosis for gliomas. *IDH1/2* mutations dominate in WHO grade II/III gliomas (also called as lower grade gliomas) and secondary GBM. According to several studies, IDH1–R132H (G395A) is the most common mutation (90%). The NOA-04 trial also showed that *ATRX* status and IDH1-R132H might be initially detected for the “integrated decision” in the further [11]. In addition, oligodendrogliomas have the 1p/19q codeletion and *TERT* promoter mutations phenotype, while *TP53* and *ATRX* mutations predominantly occur in grade II/III astrocytoma and secondary GBM. Maybe, oligoastrocytomas (WHO grade II/III) may be separated into two groups, genetically matching oligodendroglioma on one and astrocytoma on the other side based on the molecular information, for example, *IDH1/2*, 1p/19q and *ATRX* and so on [27, 28]. The frequency of *IDH* mutations is rare in primary GBMs that have *TERT* promoter mutations, *EGFR* alteration and *PTEN* loss. Yao et al. analyzed *IDH1* and *IDH2* status at codon 132 of *IDH1* and codon 172 of *IDH2* by direct sequencing and anti-IDH1-R132H immunohistochemistry in 53 paired samples and their recurrences [29]. We also found that IDH1-R132H and ATRX expression status of the recurrent tumor was most consistent with that of their initial tumor and that IDH1-R132H positive gliomas had a significantly longer progression-free survival (PFS). In addition, our cohort contained a larger sample size, thus, we assessed the diagnosis value IDH1-R132H and (or) ATRX status for human gliomas and observed that the progressive pattern of gliomas based on the precious cohort [2, 11, 30–32]. The “Haarlem Consensus Guidelines for Nervous System Tumor Classification” [10] suggest that some entities will require molecular information to provide an “integrated” diagnosis, which is based on several layers comprising (i) the integrated diagnosis as top layer, followed by (ii) histological classification, (iii) WHO grade, and (iv) molecular information. In the present study, for the first time to our knowledge, we illustrated an evaluation formula for the evolution of gliomas by IDH1-R132H combined with ATRX immunohistochemistry and identified the association between IDH1-R132H/ATRX loss and longer progression time interval of gliomas. In addition, we also observed that most recurrences had a consistent IDH1 and ATRX status with their matched primary tumors and demonstrated the progressive pattern of grade II astrocytoma/oligodendroglial tumors and anaplastic oligoastrocytoma with or without IDH1-R132H.

## MATERIALS AND METHODS

### Patients and samples

211 serial sampling of glioma pairs were obtained from the Chinese Glioma Genome Atlas (CGGA), including 202 patients with primary and the first recurrent tumors, 8 patients with primary and two times recurrent tumors and 1 patient with primary and three times recurrent tumors. Tumor tissue samples were obtained by surgical resection during January 2008 through March 2015. The histological diagnoses were confirmed by two neuropathologists according to the 2007 World Health Organization (WHO) classification guidelines [1]. The distinction between primary and secondary GBM was based on the presence of prior specimens showing lower grade gliomas. All patients provided written informed consent, and the study was approved by the ethics committees of the participating hospitals. Patients' progression-free survival data were recorded when the relapse occurred. Patients who underwent biopsy alone or lost follow-up were excluded from the survival analysis.

### Immunohistochemistry for IDH1-R132H and ATRX

Immunostaining was performed according to the manufacturer's protocol. In brief, formalin-fixed, paraffin-embedded tissue sections cut to four micrometer were dried at 80°C for 15 min and dewaxed in xylene, rinsed in graded ethanol, and rehydrated in double-distilled water. The sections were then treated with 3% H_2_O_2_ for 5 min at room temperature (RT) to block endogenous peroxidase activity. For antigen retrieval, slides were pretreated by steaming in sodium citrate buffer (10 mM sodium citrate, pH 6.0) for 15 min at 100°C. After washing with phosphate-buffered saline for 3 min, the sections were immunostained with an anti-human IDH1-R132H antibody (at 1:60 dilution, H09, Dianova, Hamburg, Germany) or an anti-human ATRX antibody (at 1:800 dilution, ab97508, Abcam), and incubated at 4°C over night. After washed by 3 changes of PBS buffer, the tissues were covered by anti-mouse/rabbit polymer HRP-label for 30 min at RT. Staining reaction was performed through covering tissue by prepared DAB chromogen solution, and incubating approximately for 10 min to allow for proper brown color development. Standard of IDH1-R312H staining: (1) a strong cytoplasmic immunoreaction product was scored positive; (2) a weak diffuse staining and staining of macrophages were not scored positive [33]. Standard of ATRX staining according to German Cancer Research Center (DKFZ) [11]: nuclear ATRX loss was scored as specific if tumor cell nuclei were unstained while nuclei of non-neoplastic cells such as endothelia, microglia, lymphocytes and reactive astrocytes were strongly positive.

### Statistical analysis

Receiver operating characteristic (ROC) curves were constructed to determine the discriminatory capacity of IDH1-R132H and (or) ATRX loss for diagnosis. Kaplan-Meier analysis was performed to estimate the survival time of different subgroups and a log-rank test was used to test prognostic differences. Comparisons of binary and categorical patient characteristics between subgroups were performed by the use of the Fisher's exact test All statistical computations were performed with the statistical software environment R version 3.2.0 (http://www.r-project.org/), GraphPad Prism Version 6.01 or Microsoft Excel 2013. *P* value < 0.05 was considered statistically significant.

## SUPPLEMENTARY MATERIALS TABLE


