# Supplementary material for: Detection of ATRX and IDH1-R132H immunohistochemistry in the progression of 211 paired gliomas
Source: Oncotarget. 2016 Feb 24;7(13):16384–95. doi: 10.18632/oncotarget.7650 (PMC4941322; doi:10.18632/oncotarget.7650)
Supplement: Supplementary file 1 [file oncotarget-07-16384-s001.pdf]

Detection of ATRX and IDH1-R132H immunohistochemistry in the progression of 211 paired gliomas

Supplementary Materials

Supplementary Table S1: Basic clinical feature for the classification of lower grade gliomas based on IDH1-R132H and ATRX status and GBM

Variables		IDH1-R132H/ATRXloss	IDH1-R132H/ ATRXexpr	IDH-WT	GBM
Age	≤ 45 years	65	44	50	100
	> 45 years	17	16	37	81
Gender	Male	45	34	46	97
	Female	37	26	41	84
Histology	A (II/III)	44	6	41	
	OA (II/III)	36	42	39	
	O (II/III)	2	12	7	
PFS	Median	860 (114–2216)	792 (105–1883)	477 (93–1771)	395 (114–2265)

Abbreviations: expr, expression; PFS, progression-free survival; A, astrocytoma; O, oligodendroglioma; OA, oligoastrocytoma; GBM, glioblastoma; IDH, isocitrate dehydrogenase, ATRX, alpha-thalassemia/mental retardation, X-linked.
